# Against Ulysses contracts for patients with borderline personality disorder

**DOI:** 10.1007/s11019-020-09967-y

**Published:** 2020-07-16

**Authors:** Antoinette Lundahl, Gert Helgesson, Niklas Juth

**Affiliations:** 1 Norra Stockholms Psykiatri, S:t Görans Sjukhus, 112 81 Stockholm, Sweden; 2 Stockholm Centre for Healthcare Ethics (CHE), Karolinska Institutet, LIME, 171 77 Stockholm, Sweden

**Keywords:** Ulysses contract, Borderline personality disorder, Autonomy, Authenticity, Decision competence, Ethics, Psychiatry

## Abstract

Patients with borderline personality disorder (BPD) sometimes request to be admitted to hospital under compulsory care, often under the argument that they cannot trust their suicidal impulses if treated voluntarily. Thus, compulsory care is practised as a form of Ulysses contract in such situations. In this normative study we scrutinize the arguments commonly used in favour of such Ulysses contracts: (1) the patient lacking free will, (2) Ulysses contracts as self-paternalism, (3) the patient lacking decision competence, (4) Ulysses contracts as a defence of the authentic self, and (5) Ulysses contracts as a practical solution in emergency situations. In our study, we have accepted consequentialist considerations as well as considerations of autonomy. We conclude that compulsory care is not justified when there is a significant uncertainty of beneficial effects or uncertainty regarding the patient’s decision-making capacity. We have argued that such uncertainty is present regarding BPD patients. Hence, Ulysses contracts including compulsory care should not be used for this group of patients.

## Introduction

Patients with borderline personality disorder (BPD) often raise distress and concern among caregivers in psychiatry (Linehan 1993; Lundahl et al. 2018). Borderline patients often experience relentless crises and display rapid changes in emotions and attitudes, due to the low tolerance for adverse situations and inner unpleasant emotions, which are characteristics of the disorder (Linehan 1993; American Psychiatric Association 2013). Moreover, suicidal and self-destructive behaviour is typically used as a means of regulating emotions and communicating inner discomfort (Linehan 1993; Black et al. 2004; Brown et al. 2002). Inpatient care is commonly applied due to suicidal behaviour, but experience has shown uncertain or even negative effects of such measures when it comes to preventing suicide and self-harm (Paris 2004; Krawitz et al. 2004; National Institute for Health and Care Excellence 2009). Therefore, inpatient care for longer than a few days has been advised against in several clinical guidelines (National Institute for Health and Care Excellence 2009; Australian Government, National Health and Medical Research Council 2012). Compulsory care, even during a crisis, is also advised against since it may inadvertently undermine the patient’s capacity to care for herself (National Institute for Health and Care Excellence 2009).

Recently, it has been shown that compulsory inpatient care on the patient’s demand occurs for this group of patients (Lundahl et al. 2017, 2018). In such cases, the presently decision-competent borderline patient usually conditions her hospital admission on attaining compulsory care—or else, the patient claims, she will not be able to withstand suicidal impulses in the imminent future. This type of compulsory care on the patient’s demand can be apprehended as a form of Ulysses contract, i.e. the patient asks to be constrained in order not to give in to future self-destructive impulses. The plausibility of such contracts has been discussed in academic and clinical circles on and off since the 1980s, especially for patients with bipolar or addictive disorders (Bell 2015), but never for patients with BPD.

In this article, we will scrutinize the arguments in favour of the use of such Ulysses contracts in health care. Our conclusion is that the arguments favouring Ulysses contracts are not solid enough to justify its use when applied as compulsory care of BPD patients.

## Ulysses contracts in health care

Ulysses contracts refer to the story of The Odyssey by Homer, in which Odysseus (Ulysses in Latin) wanted to experience the pleasure of the luring song of the lethal Sirens but at the same time be hindered from giving in to their calling. Thus, he stuffed the crew’s ears with wax and told them to tie him to the mast, so that he may not break free during the passage of the Sirens even if he begged to be released. In parallel, voluntary contracts limiting the patient’s future freedom have been advocated in health care, particularly in the treatment of bipolar disorder and substance use disorder (Bell 2015; Culver and Gert 1981; Howell et al. 1982; Macklin 1987; Elster 2000). These contracts are thought of as pre-emptive agreements to treatment and detention, and are to be implemented under certain conditions specified in the contracts; for instance, when a drug addict wants to give in to her cravings or a bipolar patient begins to show manic symptoms. The contracts are usually meant to be enforced regardless of the patient’s anticipated resistance, in a future situation when the patient is most likely legally competent. Other terms for “Ulysses contract” have been used in the academic discussion, for instance “psychiatric will” and “binding voluntary commitment” (Szasz 1982; Howell et al. 1982), but in this article we will keep to the “Ulysses contract” term (Bell 2015; Culver and Gert 1981).

When Ulysses contracts were first discussed in the 80s, it was regarding the treatment of recurrent manic episodes (Culver and Gert 1981). The argument was that patients with such disorder should not be refused to make prearrangements on how to be treated when in a future psychotic state; that refusal from the caregiver to establish such contracts would be a paternalistic infringement on the patients’ liberties (Howell et al. 1982). Others saw Ulysses contracts as a way of providing the patient with the benefits of involuntary psychiatric treatment, but without the use of formal compulsory care (Szasz 1982). These Ulysses contracts were to be formulated when the patient was deemed competent to make decisions concerning her own welfare and applied when that competency was lost due to mental illness (Szasz 1982). Apart from bipolar disorder, Ulysses contracts have also been advocated in the treatment of substance use disorders (Elster 2000; Schelling 1992). Unlike the manic state of bipolar disorder, patients with substance use disorder are generally not considered legally or mentally incompetent (Valverde 1998).

However, the legal status of Ulysses contracts, and the broader category of advance directives, is somewhat murky (Macklin 1987; Dresser 1984), so, for instance, the treating physician in the US still has the authority to override the contract when considered necessary in emergency situations (National Alliance on Mental Illness 2019). One early legal argument against advance directives was the inherent problem with making an initial consent absolutely binding in a later state when the patient resists the intervention (Macklin 1987; Dresser 1984). Deeming the individual’s prior wishes as more valid than her present wishes is legally dubious in many legislative areas (Macklin 1987; Dresser 1984). As a consequence, health care personnel could stand the risk of being liable for whichever path they take, whether it is action or inaction (Chodroff and Peele 1983). This legal minefield could be one of the reasons why Ulysses contracts and other advance directives have not been implemented as completely legally binding in the US, but rather as advisory documents.

Despite the legal problems and limitations, there have been several ethicists and clinicians endorsing the implementation of Ulysses contracts in the last two decades (Bell 2015), based on arguments ranging from weakness of will (motivating the need of external force not to give in to temptation) (Elster 1984, 2000) and autonomy as authenticity (that a patient’s long-held desires, based on her deeper values, should override deviating short-term desires) (Macklin 1987; van Willigenburg and Delaere 2005; Andreou 2008) to lack of free will (since we are all slaves under neurochemical processes in the brain) (Sedgwick 1993). These arguments have been thoroughly summarized in an article about Ulysses contracts in the treatment of substance use disorder, written by Kristen Bell (2015). In this article we will draw on her set of arguments, applied to the treatment of BPD.

## Ulysses contracts in the treatment of patients with BPD

When we look at the arguments pro and contra Ulysses contracts in substance use disorder (Bell 2015), we find that many can be easily transferred to BPD. Concurrently, patients with BPD are generally considered decision competent, and even if this competency can be diminished in moments of crisis, it is usually not as reduced as to deem the patient decision incompetent (Little and Little 2010; Owen et al. 2008; Pickard 2011; Ayre et al. 2017; Szmukler 2009). BPD patients also experience recurrent impulsive urges that can entail dangerous consequences (Linehan 1993). These impulsive urges comprise actions of self-harm, suicidality, violence, and substance misuse (Linehan 1993; Black et al. 2004; Brown et al. 2002). Borderline patients usually have insight into their problem, are capable of reasoning and making decisions without interference of psychotic delusions or thought disorder, and are able to change their behaviour in relation to personal treatment and psychotherapeutic interventions (Linehan 1993; American Psychiatric Association 2013; Little and Little 2010; Pickard 2011; Ayre et al. 2017; Dawson 1993). However, when in crisis they can find it difficult to withstand short term destructive “solutions” to their inner discomfort (Linehan 1993). Accordingly, they are in many ways similar to addiction patients, for whom Ulysses contracts have been discussed since long: they are generally considered to be decision competent (in a standard meaning of the term—see more below) (Hoge et al. 1997; Applebaum and Grisso 1995), but have difficulties resisting impulses or cravings to use potentially dangerous substances. In this article, however, we will not scrutinize the arguments concerning Ulysses contracts for patients with other psychiatric disorders than BPD, and we do not take a stand on whether the use of Ulysses contracts is justified for patients with other diagnoses.

In two previous articles—one smaller quantitative study and one qualitative study, both conducted in Stockholm, Sweden (Lundahl et al. 2017, 2018)—it has been shown that patients with BPD sometimes seek help, for instance, in emergency units, but then condition their hospital admission to being under compulsory detention, since they fear not being able to withstand destructive impulses in the near future. Thus, compulsory care in these cases becomes a form of Ulysses contract, even though this form of care has no support in the Swedish Mental Health Act. It is not known to what extent this type of compulsory care on the patient’s demand occurs, but the phenomenon has been recognized by most psychiatrists involved in the studies. The reason for this behaviour has not been fully researched, but harmonizes with the description of active passivity, a behavioural trait commonly associated with BPD (Linehan 1993). Linehan (1993) describes this as “the individual is active in trying to get others to solve her problems or regulate her behaviour, but passive about solving problems of her own”. Theories about underlying causes point at combinations of temperamental disposition (high autonomic reactivity), history of failing attempts to control negative affections and consequent maladaptive behaviours, non-validating responses from the environment, and a sex-role stereotypical interpersonal interaction style (Linehan 1993).

It is not difficult to see how Ulysses contracts come into question with borderline patients. Like Ulysses, they are well aware of the potential danger that waits ahead, they have little faith in their ability to hold back impulses to give in to future hazardous but anxiety-reducing actions, and they seek relief from conflicting wishes by transferring responsibility of their actions to a protecting crew.

## Arguments supporting the use of legally binding Ulysses contracts in treatment of BPD

It is common for patients to make voluntary advance agreements with their caregivers about restrictions concerning leaves from hospital and other interventions when the patient experiences a crisis. However, such interventions depend upon the patient’s cooperation and willingness to comply without the use of coercion or violence and are therefore not Ulysses contracts in the sense we discuss here. Such voluntary interventions will hence not be scrutinized in this paper. Instead, we will evaluate the arguments that have been presented in favour of adopting Ulysses contracts in form of compulsory care and investigate to what extent they could be considered valid when applied to the case of BPD patients. As a point of departure we will accept both consequentialist considerations of beneficence and harm-reduction as well as considerations based on autonomy. The question we discuss is if considerations of these kinds can sufficiently buttress the suggestion to implement Ulysses contracts for BPD patients. Another point of departure is that compulsory care can be justifiable, for instance and primarily when a patient lacks decision competence concerning the care offered and, hence, is unable to make an autonomous decision, and the care is considered beneficial for the patient in question. That is, we accept weak paternalism and, accordingly, our criticism against Ulysses contracts for BPD patients does not rest on a dismissal of compulsory care in general. However, since depriving a decision competent person of her freedom involves serious disrespect of her autonomy and dignity, uncertainty of whether the patient suffers from decision incompetence, or uncertainty whether compulsory care unequivocally favours the patient, will not be accepted as sufficient arguments to detain a patient under compulsory care—even if the patient pre-emptively agrees to it. There must be sufficient reason to believe that the patient is decision incompetent during the assessment under the Mental Health Act, and that the care is beneficial to the patient, in order for compulsory care to be justified—regardless of Ulysses contract, or so we will argue.

The arguments, again, are found in the academic debate from the last decades (Bell 2015). More specifically, the arguments related to autonomy concern: (1) lack of free will, (2) self-paternalism, (3) lack of decision competence, and (4) the authentic self. The argument related to consequential ethics is: (5) a practical solution in emergency situations. In the following, we will evaluate the arguments.

### Lack of free will

Many scholars refer to neurobiological research when arguing in favour of Ulysses contracts (Little and Little 2010; Carter et al. 2012; Caplan and Arthur 2008). In short, the argument goes that since everything we think or do is a result of neurobiological processes, which are beyond our mental control, we are governed by our neurobiological setup and lack free will. In neurocognitive studies, patients with BPD display a heterogeneous array of subtle abnormalities, such as deficits in attention, memory and executive functions—such as poor/risky decision making and planning (Dell’Osso et al. 2010). The deficits in decision-making may be related both to their behavioural traits, such as affective dysregulation and impulsivity, and to proposed neurocognitive dysfunctions (Dell’Osso et al. 2010). However, although neuropsychological testing appears to be sensitive to the neurocognitive deficits of BPD, the clinical utility of these results is limited (Ruocco 2005). Nevertheless, these neurocognitive abnormalities could entertain the notion of BPD patients being victims of their neurobiology and unable to make another choice than what their neurobiological setup has determined. Using force to protect a person from self-destructiveness can therefore be perceived as protecting the person from her own “faulty” neurobiology. This would justify Ulysses contracts and compulsory care with the argument that the BPD patient’s neurobiological setup makes her non-autonomous when in crisis, and that compulsory care is the only way to restore autonomy. We suggest that this line of reasoning is problematic on three fronts.

Firstly, we do not question that all behaviours correlate to some neurobiological process in the brain, as do our personality traits and emotional reactivity. However, it is well-known that the question of free will is under constant dispute, as is the question whether or not causally determined events in the brain (if there are such—we remain agnostic) cause our actions and thus make our actions incompatible with free will (Jeppsson 2012).

Secondly, even if humans lack free will, this does not necessarily confer inability to act autonomously. One can choose a way of describing autonomy that does not postulate free will: Acting autonomously in a situation only means doing what one has decided to do (following one’s own decision) and deciding to do what one wants to do (following one’s own desire)—no matter what controls our will, like neurobiology or experiences. Thus, a necessary condition for autonomy is that the desires give rise to the decision and the decision gives rise to the action (Beauchamp and Childress 2013). In fact, no stronger conception of autonomy is usually presupposed in bioethics (DeGrazia 2005; Beauchamp and Childress 2013). From this perspective there is reason to presume that BPD patients’ actions are autonomous in situations when Ulysses contracts come into question. This harmonises with the clinical experience of BPD patients’ generally good intellectual abilities, decision competence, and susceptibility to reasoning (Linehan 1993; Little and Little 2010; Pickard 2011).

Thirdly, the argument does not distinguish between BPD patients and fully healthy individuals the way it is intended to; not only BPD patients would be victims to their neurobiology if a person’s neurobiology completely determines her choices, which means that the argument opens up for Ulysses contracts, and compulsory care, also for individuals untroubled by psychiatric illness, if desired effects on health or wellbeing are achieved. To avoid this problem by insisting on a division between BPD patients and those in full health regarding neurobiology would be absurd. It would be an odd universe indeed if the will and choices of psychiatric patients in general were causally determined by their neurobiology, while those of other people were not.

### Self-paternalism

One can argue that the Ulysses contract, in the form of compulsory care, comprises too much paternalism. However, this paternalism is chosen by the patient herself, making it a form of self-paternalism. No doubt, this sounds far more attractive than external paternalism, if autonomy is considered important. Also, allowing self-paternalism can even be seen as a means of empowering the patient by letting her decide on future infringements of her own liberty.

In spite of the seeming touch of reasonableness, self-paternalism is still a form of paternalism—in our case probably strong paternalism (Beauchamp and Childress 2013), since if applied as compulsory care, the caregiver is expected to override decisions of a person who probably acts autonomously, i.e. with decision competence (Little and Little 2010; Owen et al. 2008; Pickard 2011). Even if the patient agrees to this paternalism initially, the Ulysses agreement requires the caregiver to exert this paternalism in a future situation when the patient has changed her mind. This paternalism can entail not only detention of various length but physical restraints and forced medication—actions for which the caregiver is both medically and legally responsible, also in situations when the patient previously agreed to them. Thus, from the viewpoint of respecting the patient’s (present) autonomy as well as the caregiver’s professional integrity, there are strong arguments against Ulysses contract-mediated self-paternalism, manifested as compulsory care. Ulysses contracts may, however, be applicable under the argument of self-paternalism in other psychiatric conditions, where illness episodes confer loss of decision competence—for example, manic states in bipolar disorder.

To still justify Ulysses contracts motivated by self-paternalism, the expected consequences for the patient’s well-being would have to be significantly positive. After all, paternalism is wielded with the purpose of benefitting the patient. However, the benefits of in-patient care of BPD patients are quite uncertain, perhaps even negative, as is overtaking the patient’s autonomy (Linehan 1993; Paris 2004; National Institute for Health and Care Excellence 2009). As stated in the NICE guidelines on treatment of BPD:

People with BPD often find it hard to cope at times of crisis, and may look to others to take responsibility for their needs. While service providers may feel under pressure to try to do this, this approach may inadvertently undermine a person’s limited capacity to care for themselves. It is therefore important to try to ensure that people with BPD remain actively involved in finding solutions to their problems, even during crises. (National Institute for Health and Care Excellence 2009)

Thus, when the caregiver takes over decision-making and agency from the patient, this impedes the patient’s learning of how to cope with her emotions, making her more vulnerable to future crises (Linehan 1993; National Institute for Health and Care Excellence 2009). There is also clinical experience of increase in regressive and self-destructive behaviour during in-patient care (Paris 2004; Dawson 1993). The expected benefit of this Ulysses contract intervention is thus not great enough to justify setting the patient’s autonomy aside.

### Lack of decision competence

The typical situation when Ulysses contracts come into question for BPD patients, is at the emergency unit, where the patient claims to be unable to accept voluntary care, since she fears not being able to resist self-destructive impulses in the near future unless she is prevented by compulsory detention (Lundahl et al. 2018). However, in the moment when the patient demands such compulsory care, she is decision competent. The patient’s indirect way of seeking help, can be understood as an expression of active passivity and lack of self-trust (Linehan 1993), common thought patterns of BPD patients in crisis, but can also be interpreted as her being in a state of fluctuating decision competence due to high levels of emotional distress (Lundahl et al. 2018). In addition, some clinicians interpret BPD patients in crisis as suffering from severe co-morbid mental disorders, motivating compulsory care (Lundahl et al. 2018; Dawson 1993).

When it comes to decision competence and psychiatric disorders, the MacArthur Treatment Competency Study in the 1990s found that the majority of patients with schizophrenia and depression were decision competent concerning psychiatric and medical treatment (Hoge et al. 1997). The study also found that decision incompetence was correlated to disorganized thought processes rather than delusions or hallucinations alone (Hoge et al. 1997; Applebaum and Grisso 1995). Thus, severe mental illness by itself does not prove the patient decision incompetent in matters of psychiatric treatment. Assessment of mental capacity has also been conducted on patients admitted to psychiatric wards and emergency units, and although there were small samples, the results endorse the notion of BPD patients generally being decision competent (Owen et al. 2008; Szmukler 2009). From a moral and legal point of view, patients are assumed to be decision competent regarding treatment options unless the caregiver can prove they are not (Hubbeling 2014). This assumption implies that when the decision competence is marginally decreased, the patient is to be treated as having decision competence (Ayre et al. 2017). The assessment of decision competence should also be independent of the consequences of the decision (Hubbeling 2014).

Commonly, BPD patients in crisis display a transient high level of emotionality and self-destructive impulses, but are also receptive to reasoning and psychological interventions, in a manner that indicates organized thought processes (Linehan 1993; Pickard 2011; Dawson 1993). This corresponds with the common opinion in psychiatry today, that BPD patients generally are decision competent when it comes to treatment decisions (Little and Little 2010; Owen et al. 2008; Pickard 2011; Hubbeling 2014). It has been argued that the decision making capacity of suicidal BPD patients in crisis could be partly impaired, for example due to pathological values, and that the caregiver should “play for time” in such situations by making time consuming assessments of capacity or consider treatment under the Mental Health Act (David et al. 2010, Hubbeling 2014). Such arguments in favour of deeming the patient decision incompetent seem based on the idea that assessing the patient as incompetent could be life-saving. However, as of yet there is no support for the claim that BPD patients are decision incompetent just because they are in a crisis or suicidal, or that BPD patients benefit from being deemed decision incompetent in such situations (Ayre et al. 2017, Little and Little 2010; Owen et al. 2008; Pickard 2011; National Institute for Health and Care Excellence 2009). Even if the patient has recurrent strong impulses to act self-destructively, high levels of emotionality, or shifting motivation to participate in psychiatric treatment, this does not necessarily render the patient sufficiently cognitively affected as to deem her decision incompetent when it comes to accepting or rejecting the care offered. Also, an apprehension of future perilous behaviour or expected inability to comply with voluntary care does not prove that this will actually happen or that the patient cannot be persuaded to accept voluntary care by less paternalistic means than compulsory care.

Taken together, even if BPD patients in crisis fear not to be able to participate in voluntary care, and even if they suffer from a severe comorbid mental illness, there is doubt to whether they objectively lack decision competence when it comes to psychiatric or medical treatment. This doubt leaves the argument of lacking decision competence too weak to motivate Ulysses contracts in form of compulsory care.

### The authentic self

Another kind of argument that has been used to endorse Ulysses contracts is that they express our authentic desires. The idea of authenticity is often expressed in ordinary language in terms of what someone “really wants”, in contrast to for that person non-typical urges or “choices out of character”, possibly explained by external pressure or “loss of contact with one’s true self”. The underlying idea is that our authentic desires (i.e. long-held desires, which are aligned with our deeper values), are more autonomous than inauthentic desires (often more short-term desires, deviating from the long-held ones). Thus, the Ulysses contract represents the person’s authentic desires, as expressed when the contract was written, and is to be implemented in a situation when the person expresses deviating, inauthentic desires (van Willigenburg and Delaere 2005). In addition, the authentic desires are often presumed to be beneficial and rational, while the inauthentic desires are considered irrational and controlled by temptations and destructive urges (Macklin 1987). Taken together, the Ulysses contract exerts the will of the patient’s authentic self and protects her autonomy against destructive, inauthentic, impulses.

Our first criticism of this argument relates to the difficulty of knowing what constitutes a person’s authentic desires. One can of course presume that the patient’s authentic desires are always those expressed in a Ulysses contract, but this seems to presuppose what must be demonstrated. In fact, it seems difficult to know which of two conflicting desires that is more authentic, especially with enough certainty as to motivate compulsory detention or other important health care decisions against the patient’s presently expressed will. Any expressed desire can be more or less authentic, on a theoretical scale, leaving the caregiver with the challenging task of deciding which desire is inauthentic enough as to motivate infringements on the patient’s liberty.

The difficulty of determining which of two conflicting desires that is more authentic is buttressed by the fact that we do change our minds about what we want and value. As humans we change, our objectives and desires differ over time (as do our neurobiology), and we sometimes change our minds concerning previous decisions. Unless we accept that transformed desires can be as authentic as the previous desires, people would risk not having their will respected when changing their mind. Correspondingly, if compulsory care is implemented based on authentic desires, as previously expressed in a Ulysses contract, the patient risks becoming a prisoner of her previous self and not having her will respected by health care, even if she is presently decision competent.

Secondly, there is no support for the assumption that destructive or impulsive desires are irrational or inauthentic, merely by being destructive and impulsive. Hence, self-damaging desires cannot be judged as any less authentic than the self-promoting ones although, of course, acting on them has worse consequences for the individual. It may be tempting to transform a basically paternalistic attitude (“she ought to be compelled not to act against her own best long term interest”) to something aligned with the patient’s autonomy (“she ought to be compelled since acting against her long term interest is not what she *really* wants”), but this is, we conjecture, nothing but a rationalization. In other words, the tail of consequence wags the dog of authenticity.

These arguments have been elaborated in previous work in the field of bioethics, arguing against compulsory care being justified by respect for authenticity (Sjöstrand et al. 2014). Unless one has firm grounds for saying that an expressed desire is not what a person “really” wants, one should abstain from doing so. In light of the difficulties of even capturing what authenticity is, the situations where the grounds are firm are rare, at best (Sjöstrand et al. 2014). In summary, the concept of authenticity is too uncertain to motivate Ulysses contracts as compulsory care for BPD patients.

### A practical short-term solution in emergency situations

There is a practical argument favouring the use of Ulysses contracts: When the patient visits the psychiatric emergency unit, expressing suicidal intentions and conditioning admission to receiving compulsory care, it might be difficult for the assessing physician to argue against compulsory care. The alternative involves a risk that the patient leaves the emergency room and self-harms or even makes a suicide attempt, which—even if the reasons are instrumental and death not intended—may lead to the patient’s demise. If this happens, the physician’s action is immediately scrutinized and questioned by health care management and authorities, as to why the patient was not compulsorily detained (Gutheil 2004). Arguing with the patient to accept voluntary admission also takes time and might result in the patient changing her mind and demanding to be discharged during on call hours, causing even more work for the caregiver (Lundahl et al. 2018). If the patient is unknown to the assessing clinician, there can also be diagnostic difficulties, leaving the clinician uncertain of whether the patient is suffering from a co-morbid severe mental illness that renders her decision incompetent. From this perspective, some clinicians argue that “it is better to be safe than sorry”, implying that compulsory care is the safe option (for both the patient and the caregiver) in emergency situations—irrespective of decision competence (Hubbeling 2014). Hence, the practise of short term Ulysses contracts in form of compulsory care in emergency situations could be justified from a consequentialist standpoint.

One argument against this conclusion is based on recent data indicating that crisis-service utilization in itself, like emergency-room visits and previous inpatient admissions, conveys risk for future suicide for patients with BPD—a negative side effect of such care (Coyle et al. 2018). Also, admission to hospital itself, unrelated to diagnosis, may play a causal role in a proportion of inpatient suicides (Large et al. 2017). Coercive measures and loss of social context during inpatient treatment have been mentioned as possible contributing factors (Large et al. 2017). Even though these two latter studies (Coyle et al. 2018; Large et al. 2017) do not specifically study the effects of compulsory care of BPD patients, they still point to possible negative effects of such care since compulsory admission signifies inpatient care in our case. Another argument is the risk of inadvertently increasing the suicide risk in the longer term by decreasing the patient's capacity to manage her own risk (National Institute for Health and Care Excellence 2009; Coyle et al. 2018). If the patient is not trusted with voluntary admission, she will not be able to confront and challenge her fears and self-doubt—denying her an opportunity to develop her skills to cope with present and future challenging situations (many BPD patients experience relentless crises over a long period of time). This will render the patient feeling more dependent and helpless in the long run. Giving in to the patient’s demand for compulsory care under the threat of potential self-harm or suicide can also enforce this type of communicative behaviour, i.e. using suicidality as a means of, for example, obtaining help or expressing inner discomfort. Enforcing this type of instrumental suicidal communication and action, increases the probability of such communication in the future, in accordance with psychological principles of behavioural change (Sundel and Sundel 2017), which inadvertently may increase the risk of suicide in the long run. Then, there is no evidence to support that hospital admission protects against suicide for patients with BPD (Paris 2004; Krawitz et al. 2004; McGirr et al. 2007). In the light of these latter arguments, it might be the patient who draws the shortest straw by receiving compulsory care in the emergency unit, while the clinician safeguards herself against potential litigations and facilitates her work. However, it is not known whether all the negative effects mentioned occur from a very short term compulsory detention (for example, until the next day).

If one argues in favour of compulsory care under the parole “better safe than sorry”, this presupposes that the consequences of compulsory care are unequivocally beneficial or at least “safe” and that the potential negative effects (the “sorry” part) only lies within abstaining from compulsory care. However, as we have just explained, it is not clear whether such intervention of short-term compulsory care is beneficial to the patient or not. And uncertainty of either consequence or decision competence, is not strong enough an argument to motivate the intervention of compulsory care—neither from a consequentialist nor from an autonomy-defending viewpoint.

## Conclusion

BPD patients are often considered difficult to treat, not the least because of their frequent mood swings, ambivalence to offered care and their unpredictable self-destructiveness and suicidality. At the same time, this group of patients is generally considered decision competent. One suggested solution to this problem is the implementation of Ulysses contracts in form of compulsory care, where the decision-competent BPD patient authorizes the caregiver to override her autonomy in order to protect her from acting self-destructively when in a crisis. In this paper we have scrutinized several arguments that have been used in previous debates concerning Ulysses contracts for other groups of patients, as well as arguments used in clinical practise of treating BPD patients. We have accepted both consequentialist and autonomy-defending considerations. We conclude that compulsory care cannot be justified when there is a significant uncertainty of beneficial effects or uncertainty regarding the patient’s decision-making capacity. We have argued that such uncertainty is present regarding BPD patients. Hence, Ulysses contracts including compulsory care should not be used for this group of patients.
